# Bacterial Communities Differ among *Drosophila melanogaster* Populations and Affect Host Resistance against Parasitoids

**DOI:** 10.1371/journal.pone.0167726

**Published:** 2016-12-14

**Authors:** Mariia Chaplinska, Sylvia Gerritsma, Francisco Dini-Andreote, Joana Falcao Salles, Bregje Wertheim

**Affiliations:** 1 Evolutionary Genetics, Development & Behaviour, Groningen Institute for Evolutionary Life Sciences, University of Groningen, Groningen, The Netherlands; 2 Genomics Research in Ecology and Evolution in Nature, Groningen Institute for Evolutionary Life Sciences, University of Groningen, Groningen, The Netherlands; International Atomic Energy Agency, AUSTRIA

## Abstract

In *Drosophila*, diet is considered a prominent factor shaping the associated bacterial community. However, the host population background (e.g. genotype, geographical origin and founder effects) is a factor that may also exert a significant influence and is often overlooked. To test for population background effects, we characterized the bacterial communities in larvae of six genetically differentiated and geographically distant *D*. *melanogaster* lines collected from natural populations across Europe. The diet for these six lines had been identical for ca. 50 generations, thus any differences in the composition of the microbiome originates from the host populations. We also investigated whether induced shifts in the microbiome—in this case by controlled antibiotic administration—alters the hosts’ resistance to parasitism. Our data revealed a clear signature of population background on the diversity and composition of *D*. *melanogaster* microbiome that differed across lines, even after hosts had been maintained at the same diet and laboratory conditions for over 4 years. In particular, the number of bacterial OTUs per line ranged from 8 to 39 OTUs. Each line harboured 2 to 28 unique OTUs, and OTUs that were highly abundant in some lines were entirely missing in others. Moreover, we found that the response to antibiotic treatment differed among the lines and significantly altered the host resistance to the parasitoid *Asobara tabida* in one of the six lines. *Wolbachia*, a widespread intracellular endosymbiont associated with parasitoid resistance, was lacking in this line, suggesting that other components of the *Drosophila* microbiome caused a change in host resistance. Collectively, our results revealed that lines that originate from different population backgrounds show significant differences in the established *Drosophila* microbiome, outpacing the long-term effect of diet. Perturbations on these naturally assembled microbiomes to some degree influenced the hosts’ resistance against natural parasites.

## Introduction

Macro-organisms can be viewed as distinct ecosystems, in which numerous microorganisms establish close mutualist, commensal and pathogenic associations with their hosts [1]. These microbial-host associations are known to influence host fitness as well as host adaptation and evolution when the microbial components are transmitted among generations—a common feature in many microbial-host systems [2]. In particular, insects are suitable model systems to disentangle mechanisms driving the interactions between the host and its associated microbiome [3].

The insect microbiome is known to influence host phenotype in a variety of ways: through diet supplementation [4], the transmission of pathogens in insect disease vectors [5], reproductive behaviour and isolation [6] and kin recognition [7]. More specifically, *Drosophila melanogaster* Meigen (Diptera: Drosophilidae) has been used as a model to study host-microbiome interactions since the beginning of the 20^th^ century and was the first gnotobiotic organism to be cultured (i.e. under aseptic conditions) [8]. Curing *Drosophila* from its microbiome revealed modifications in a number of host physiological responses, ranging from reproduction [9] to immunity and resistance to parasitoids and pathogens [10–15]. Some of these changes are attributed to the genus *Wolbachia*, a widespread endosymbiont of arthropods [9], but *Wolbachia* is not the only bacterium affecting hosts’ fitness. Also gut-associated bacteria [16], other endosymbionts [15], or microbes that reside on its exogenous body parts [17] can exert great influence on their host, by affecting lifespan [17], intestinal stem cell activity [18], kin recognition and mate choice [7,19]. Although much research has been performed on the range of processes that are affected by the microbiome [20], it is still not clear what the relative contributions are of different mechanisms shaping the *Drosophila* microbiome and host-symbiont interactions.

Despite its relative simplicity, consisting of 1–30 OTUs [21,22] and usually dominated by 1 or 2 taxa [22,23], the *Drosophila* microbiome is dynamic, changing throughout the developmental stages of the host [21]. The factors that are most likely to exert an influence on the establishment of *Drosophila* microbiome have been shortlisted: host diet [23,24], host taxonomy [23,25], geography, morphology, genetics, physiology [26], random events [24] and surrounding environmental conditions [27]. Different studies have been using different methods, so comparisons should be done with caution. However, the debate persists on which factors have a dominant role. Whereas some studies suggested the core *Drosophila* microbiome to be shaped by the diet, and not by geography or host taxonomy [21,23], others established that taxonomy [25] or stochastic processes, rather than diet, was the major driver of bacterial composition and disputed the existence of a core microbiome in *Drosophila* [21,24].

Population background is also a factor that can potentially exert an influence on the *D*. *melanogaster* microbiome. For instance, it was shown that the microbiome of freshly caught *D*. *melanogaster* flies from various fruits across distant geographical populations differed in bacterial composition [28]. Natural populations encounter local conditions that may vary considerably, in terms of abiotic conditions (e.g. temperature, humidity), the available food sources and the other organisms that inhabit these environments. Moreover, as *D*. *melanogaster* feed and breed on a variety of decomposing fruit [29], they are exposed to a broad range of microorganisms, which form a pool of mutualists, commensals and potential pathogens [27]. It has been demonstrated that natural populations of *Drosophila* adapt to their local conditions, including differentially resisting various bacterial pathogens [26] and parasites [30,31]. The underlying mechanism for resistance against bacterial pathogens is associated with genetic variation in immunity genes [26]. Since interactions between fruit fly hosts and microorganisms can vary greatly between different environments, this could imply that *Drosophila* populations of different genetic backgrounds are capable of acquiring and maintaining different bacterial types. One tantalizing question is whether the phenotypic and genetic variation among natural populations in parasite resistance is perhaps partially mediated by the complex interactions between the host and its microbiome.

This study aimed to determine whether population background affects the *Drosophila* microbiome in natural populations reared in the lab under identical conditions for over 4 years, and whether the established *Drosophila* microbiome is of significance for host immunity against parasites. Firstly, we determine whether six genetically differentiated populations of *D*. *melanogaster* differ in microbiome when controlling for diet effects for ca. 50 generations. If host population origin (and not only diet) plays a role in shaping the bacterial community, we would predict differences between microbiome compositions among different *Drosophila* lines that were reared on identical diets for years. Population background differences may reflect the population genetics of the lines, or alternatively, long-lasting associations with the original microbiome. Secondly, we evaluated whether antibiotic manipulation of the hosts’ microbiome had a phenotypic effect on host resistance to the parasitoid *Asobara tabida* Nees (Hymenoptera: Braconidae). *Asobara tabida* is a small wasp, that attacks 2-3^rd^ instar larvae of *Drosophila* and lays an egg, which will either develop into an adult wasp and kill the fly, or will be killed itself by the hosts immune response [32–36]. Natural populations of *D*. *melanogaster* vary in their resistance to *A*. *tabida* [31,32]. If the variation in the resistance is mediated by host-microbiome interactions, we would predict changes in parasitoid resistance after *Drosophila* microbiome was altered by antibiotics. Finally, if the microbiome differs among lines of different population backgrounds, we might expect dissimilar effects of antibiotic treatment on parasitoid resistance among those *Drosophila* lines. To test our hypotheses we assessed the bacterial community composition, diversity and abundance, as well as the specific abundance of *Wolbachia* in 3^rd^ instar larvae of six *D*. *melanogaster* lines, i.e. at the developmental stage that has to defend itself against the parasitoid. We subjected these lines to a broad-spectrum of antibiotic treatments to disturb the indigenous microbiome and tested whether this would affect their resistance to *A*. *tabida*.

## Materials and methods

### *Drosophila* samples and rearing

Six lines of *D*. *melanogaster* were collected from natural populations across Europe in 2009. These lines were established in the laboratory as mass cultures (>>1000 individuals/line/generation). After the mass cultures had been well established, we measured their resistance to *A*. *tabida* [31] and their genetic differentiation. The lines differed significantly in parasitoid resistance [31] and showed substantial genetic differentiation, as indicated by an average pair-wise F_st_ value of 0.124±0.015 (determined from a subsample of 12 females/mass culture line in 2011) [37]. The lines were established from multiple foundresses (6–60) and had been kept in the laboratory for 4 years prior to this study. Further information on fly collection, maintenance and the resistance study can be found in our previous study [31]. In short, the lines originated from: Germany (Bayreuth, BAY; Bremen, BRE), Scotland (St. Andrews, STA), The Netherlands (Groningen, GRO) and France (Gotheron, GOTH; Arles, ARL). Adult female flies were captured in traps and cultured in the lab as iso-female lines for one generation. Per locality mass cultures were established by mixing the offspring of the iso-female lines.

The insect lines were reared in 10 quarter-pint bottles containing 30 mL standard medium (26 g heat-inactivated yeast, 54 g sugar, 17 g agar and 13 mL nipagin 8.5 mM solution, solved in 1 L), at 20°C and 12h:12h dark:light regime. The offspring (>>1000) of each generation was mixed and distributed over 10 quarter-pint bottles. Larval density was standardized every generation for all field lines to avoid competition through overcrowding, and to maintain the genetic diversity in the mass cultures.

### Ethics statement

This study is exempted from institutional or national regulations on animal research, as it involves invertebrates that do not require such permission. Also the collection of *D*. *melanogaster* and their parasitoids from natural populations in Europe is not restricted by ethical approval, permissions or regulations.

### Microbiome composition

#### DNA extraction

Total genomic DNA was extracted from pooled samples containing ca. 30–40 2-3^rd^ instar *D*. *melanogaster* larvae. We collected three biological replicates per line. Larvae were not surface-sterilized because it was previously demonstrated that *Drosophila* have an exogenous bacterial community, which is also important for the host’s physiology [17]. To collect larvae, adults were incubated overnight for egg laying on Petri-dishes with standard medium at 25°C. The adults were removed in the morning and the eggs transferred to 20°C. At 72 h after egg laying the larvae were carefully collected with a fine sterile spatula and snap-frozen in liquid nitrogen.

Insects were thoroughly homogenized with a sterile motorized pestle to make sure intracellular bacterial DNA (e.g. *Wolbachia*) was also extracted. DNA was isolated using the Power Soil^®^ DNA Isolation Kit, following the manufacturer`s protocol (Power Soil^®^, MoBio Laboratories Inc., California, United States). DNA concentration was quantified using NanoDrop ND2000 (Thermo Scientific^TM^) and standardized to concentrations of 25 to 50 ng μL^-1^.

#### PCR condition for the partial amplification of the bacterial 16S rRNA gene

Partial bacterial 16S rRNA gene was PCR-amplified using the primer set F968/R1401 (S1 Table)—expected fragment size of 433 bp—in the following 50 μL master mix: 0.4 μL of 25 mM dNTPs, 3.75 μL of 50 mM MgCl_2_, 5 μL of 10x PCR Buffer, 0.5 μL of Formamide, 0.25 μL of 20 mg mL^-1^ bovine serum albumin (BSA), 200 nM of forward and reverse primers, and 0.5 μL of 10 U μL^-1^ Taq DNA polymerase (Roche Applied Science, Germany). To ensure the specificity of the reaction, touchdown PCR condition was set as follows: the initial denaturation step at 94°C for 5 min, followed by 10 cycles of 94°C for 1 min, 60°C (lowering the temperature by 0.5°C every cycle) for 1 min, 72°C for 2 min; and by 25 cycles of 94°C for 1 min, 55°C for 1 min, 72°C for 2 min; with a final step of 72°C for 30 min. The presence and specificity of the amplicons were verified in 1.5% agarose gel stained with ethidium bromide.

#### Denaturing Gradient Gel Electrophoresis (DGGE)

The DGGE analysis was performed to estimate differences in the structure of bacterial communities across populations of *D*. *melanogaster* and to determine the sampling effort needed to fully characterize their community composition. 16S rRNA bacterial genes were PCR-amplified using the primer set F968 with a GC-clamp attached to 5’ and R1401 (S1 Table), as described above. The obtained amplicons were further used for the DGGE analysis. The DGGE were visualized with Imagemaster VDS (Amersham Biosciences, Buckinghamshire, United Kingdom) and further analysed with GelCompar software (Applied Maths, Sint-Martens Latem, Belgium). The observed low bacterial diversity, as evidenced by the low number of bands on DGGE gels (S1 Fig), justifies our choice of sequencing method (Sanger sequencing) and was used to determine sampling size.

#### Cloning and sequencing of the bacterial 16S rRNA gene

The previously amplified 16S rRNA gene region (without the GC-clamp) was used for the cloning library. The PCR product was diluted and 20 ng of the amplicon was ligated into the pGEM-T vector (Promega, manufacturer’s instructions). Competent *Escherichia coli* cells were used for the transformation step. For each of the 6 lines, 3 biological replicates were used.

For each replicate ~90 clones were picked from the agar plates for sequencing and analyses. Positive clones were amplified using pGEM-T forward and reverse primers (S1 Table). The amplicons were checked for size and concentration on 1.5% agarose gel. PCR products of the expected ~440 bp were purified following the manufacturer’s recommendations (ExoSap-IT^*®*^, Affymetrix) and further used for sequencing on ABI3170 Prism sequencer by Applied Biosystems, following the manufacturer’s protocol (Applied Biosystems Big Dye^®^).

#### Analyses of bacterial 16S rRNA gene clone libraries

Obtained sequence chromatograms were initially trimmed using the Lucy algorithm [38] at a threshold of 0.002 (quality score of 27), available within the Ribosomal Database Project (RDP) pipeline (https://rdp.cme.msu.edu/pipeline/). Only sequences with trimmed lengths longer than 320 bp were retained for analysis (i.e. 1,044 sequences representing the six lines of *D*. *melanogaster*). In order to integrate the cleaned sequence data into the QIIME pipeline [39], we artificially added barcodes sequences (ca. 10 bp) at the 5’ of each sequence. Different barcodes were added for each sample. Operational taxonomic units (OTUs) were generated by binning the sequences at 97% of nucleotide identity using Uclust [40]. Selected representative sequences per OTU were aligned against the Greengenes coreset [41] using PyNAST [39], with sequences classified using the Greengenes taxonomy via RDP classifier [42]. The alignment was filtered to remove common gaps and a phylogenetic tree was constructed *de novo* using FastTree [43].

For all OTU-based analyses, the original OTU table was rarefied to a depth of 50 sequences per sample (i.e. the lowest in a single sample), to minimize effects of sampling effort on the analysis. This was carried out using default parameters in QIIME (script *alpha_rarefaction*.*py*) that conducts 10 iterations per sampling depth. Rarefaction curves and the estimated sample coverages for each sample are shown at S2 Fig and Table 1, respectively. One replicate (ARL_2) was excluded from the analysis due to the low number of sequences (ca. 30).

**Table 1:** Estimation of alpha-diversity indices from bacterial communities associated with *D*. *melanogaster* lines.

Library name	Number of sequences	Number of OTUs^1^	Chao1 index	Shannon’s index	PD^2^	ESC^3^
ARL_1	50	19	30	3.23	0.81	0.76
ARL_3	50	8	10	1.77	0.56	0.93
BAY_1	50	7	9	1.5	0.58	0.93
BAY_2	50	11	17	2.36	0.74	0.89
BRE_1	50	17	26	3.45	1.05	0.84
BRE_2	50	16	40	2.75	0.98	0.76
BRE_3	50	13	30	2.68	0.81	0.82
GOTH_1	50	9	16	1.84	0.59	0.89
GOTH_2	50	7	10	2.23	0.37	0.94
GOTH_3	50	6	7	1.25	0.38	0.95
GRO_1	50	8	10	2.24	0.43	0.94
GRO_2	50	10	16	2.58	0.5	0.9
GRO_3	50	7	7	2.03	0.36	0.98
STA_1	50	5	9	0.54	0.35	0.93
STA_2	50	4	6	0.49	0.32	0.95

Sample size, richness estimator, diversity indices and sample coverages of the microbiomes of six *D*. *melanogaster* lines. Biological replicates are indicated as numbers next to the line abbreviation: ARL, Arles; BAY, Bayreuth; BRE, Bremen; GOTH, Gotheron; GRO, Groningen; STA, St. Andrews.

^1^Calculated with QIIME at 97% nucleotide identity, at the same rarefaction depth of 50 sequences per sample

^2^Faith’s Phylogenetic Diversity

^3^Estimated sample coverage (C_x_): C_x_ = 1 –(N_x_/n), N_x_: the number of unique sequences, n: total number of sequences per sample

The QIIME was also used to generate weighted/unweighted UniFrac distance matrices [44] and alpha-diversity metrics, including OTU richness (unique OTUs), ChaoI richness estimation, Shannon’s and Faith’s phylogenetic diversity indices. Alpha diversity differences were compared among lines using an ANOVA (*group_significance*.*py* in QIIME [45]). The OTU Venn diagram was constructed using jvenn [46].

All sequencing data have been deposited in the NCBI database under submission KT189679 –KT190693 (http://www.ncbi.nlm.nih.gov/).

#### Antibiotic treatment for host microbiome manipulation

To induce a shift in the microbiome, we temporarily treated the flies with antibiotics, and then enabled re-establishment of a larval microbiome through natural colonization processes (i.e., from contact with the food and the faeces of parental flies). The *D*. *melanogaster* lines were divided into two groups: control and treatment. The control group was reared on the standard medium, while a combination of antibiotics was added into the medium of the treatment group for three successive generations [19]. The antibiotic mixture contained: rifampycin (400 μg mL^-1^), streptomycin (100 μg mL^-1^) and tetracycline (200 μg mL^-1^) (Sigma-Aldrich^®^). After the treatment, the treated *D*. *melanogaster* lines were reared for two generations on standard (antibiotic-free) medium in order to eliminate possible toxic effects of the antibiotics on the fly metabolism, and to allow for the establishment of the (shifted) microbiome.

### Parasitization experiment

We used the *A*. *tabida* strain TMS to test *D*. *melanogaster* resistance to parasitoids [47]. The parasitoid wasps were reared on a low resistant line of *D*. *melanogaster* at 20°C under a 12h:12h dark/light regime. The ability of *Drosophila* to encapsulate *A*. *tabida* eggs was used as a measure for host's resistance to parasitoids [31]. We performed the test and measured resistance in all six *D*. *melanogaster* lines. For the parasitization trial, ten 2^nd^ instar larvae and two *A*. *tabida* females were placed in a Petri dish, and the wasps were allowed to parasitize for nine hours. Each parasitization trial was replicated ten times. Once the larvae pupated, they were counted and transferred to new vials containing agar medium. Both emerging flies and wasps were counted. Flies were examined for the presence of encapsulated eggs by squashing them between two glass slides, as the melanized capsules are clearly visible this way. The encapsulation rate (ER, %) was defined as the percentage of adult flies carrying one or more capsules (c, indicating successful encapsulation) of the total parasitized individuals (p). The number of parasitized individuals was estimated as the sum of adult flies carrying an encapsulated egg (c) and the number of emerged wasps (w, indicating no or unsuccessful encapsulation). The following formula was used:

$$ER(\%)=\frac{c}{c+w}\times 100=\frac{c}{p}\times 100$$

To analyse the data from the parasitization experiment we used a generalized linear model (glm) approach implemented in R 3.0.2 [48]. To compare the encapsulation rate among the six tested lines, the per replicate data on the number of emerged adult flies with capsule (c) and the number of emerged wasps (w) were combined in a two-vector response variable (*Ratio c*:*w*). The glm models tested whether this ratio was significantly different among the lines and after antibiotic treatment. We also analysed the pupa-to-adult mortality in these experiments, combining the numbers of pupa from which an adult (wasp or fly) emerged and those that died in a two-vector response variable, and comparing this ratio among lines and after antibiotic treatment. To judge the statistical significance of explanatory factors (Line, Treatment and the interaction between Line and Treatment) on encapsulation rate, we removed the explanatory variables one by one from the maximal model and used *F*-tests for comparisons of the simplified model to the model including the explanatory variable [49]. We specified a quasibinomial distribution for the glm models to correct for over-dispersion. The quasibinomial distribution differs from the binomial distribution by scaling the dispersion parameter to align the residual deviance with the degrees of freedom, making the model more conservative and reducing the false error rate. For post-hoc analyses, including a single-step multiple testing correction, we defined a combined explanatory factor of Line and Treatment and tested within each line for significant effects of antibiotic treatment. All scripts for the statistical analyses are included in the Supplementary Material (S1 File).

### Quantitative Real-time PCR

Quantitative real-time PCR (qPCR) was performed on DNA samples, diluted to the final concentration of 20 ng μL^-1^. Three biological and two technical replicates were performed for all *D*. *melanogaster* lines and treatments (i.e. control, antibiotic treated). Per reaction, 1 μL DNA template was mixed with 12.5 μL ABgene ABsolute^TM^ QPCR SYBR^®^ Green ROX Mix (500 nM) (Thermo Fischer Scientific, Germany) and 200 nM of forward and reverse primers for TATA-binding protein (*Tbp*), 16S rRNA or *gatB* (S1 Table). Primer selection and optimisation was performed on a dilution series of cDNA templates to ensure high and similar efficiency, as well as linear amplification, for all primers sets. The *Tbp* primers were used to quantify host DNA, the 16S rRNA primer measured the total bacterial abundance, and the *gatB* primers specifically targeted a *Wolbachia* gene. *Tbp* was chosen as reference gene because of its high conservation so that the DNA primers would not be hampered by sequence variations across the natural populations. The following qPCR settings were used: enzyme activation of 95°C for 15 min, followed by 45 cycles of 95°C for 15 s, 55°C for 30 s, 72°C for 30 s (data collection point); and a final step (extension) of 72°C for 7 min. As a negative control, 1 μL of milliQ water was used instead of DNA template. Samples were checked for non-specific amplification and primer-dimers using a standard ABI7300 dissociation curve.

LinRegPCR software was used to estimate the initial concentration (C_0_) of 16S rRNA and *Wolbachia* gene copies, incorporating estimates of PCR efficiency [50]. To standardize for the differences in DNA concentration between templates, the relative abundance of 16S rRNA and the *Wolbachia gatB* genes was calculated by dividing their C_0_ (initial concentration) by the C_0_ of the reference gene (*Tbp*). To test for differences in the (standardized) abundance of the 16S rRNA gene and the *gatB Wolbachia* gene, we used a linear model with random effects approach implemented in R 3.0.2 on log-transformed data. The technical replicates per line per treatment group were analysed separately, and biological replicate was used as a random effect in the model to take the co-variation between the technical replicates into account. We then removed the explanatory variables (Line, Treatment and the interaction between Line and Treatment), one by one from the maximal model and used *F*-tests for comparisons of the simplified model, to test for differences in bacterial load among the lines, and whether *Wolbachia* infection had been cured by the antibiotics treatment prior to parasitization experiment.

## Results

### The microbiome composition of *D*. *melanogaster* lines

**Fig 1:**
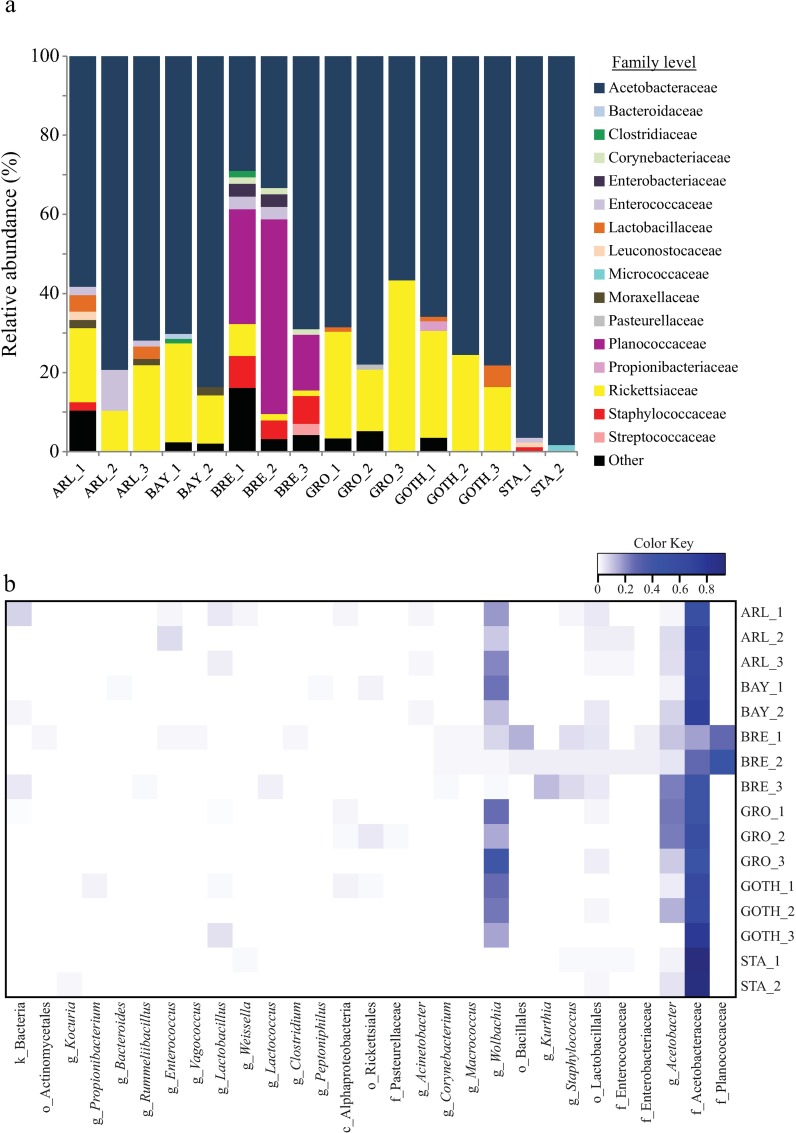
The relative abundance of bacterial taxa in six *D*. *melanogaster* lines, based on the taxonomic affiliation of a 16S rRNA gene fragment. The relative abundances of (a) bacterial families and (b) bacterial genera in six lines of *D*. *melanogaster*, which were derived from natural populations across Europe. Biological replicates are indicated as numbers next to the line abbreviation: ARL, Arles; BAY, Bayreuth; BRE, Bremen; GOTH, Gotheron; GRO, Groningen; STA, St. Andrews.

To describe microbial communities associated with *D*. *melanogaster* lines, 16 clone libraries were obtained (2–3 per line). A total of 1,044 clones were successfully sequenced. Based on 97% of nucleotide identity, the sequences were binned into 75 OTUs (S2 Table). Classification to the genus level (not possible for some OTUs) revealed the presence of 18 distinct genera (Fig 1), encompassing 42 OTUs. Three genera were represented by a minimum of 5 different OTUs: *Acetobacter*, *Staphylococcus* and *Wolbachia* (S2 Table).

**Fig 2:**
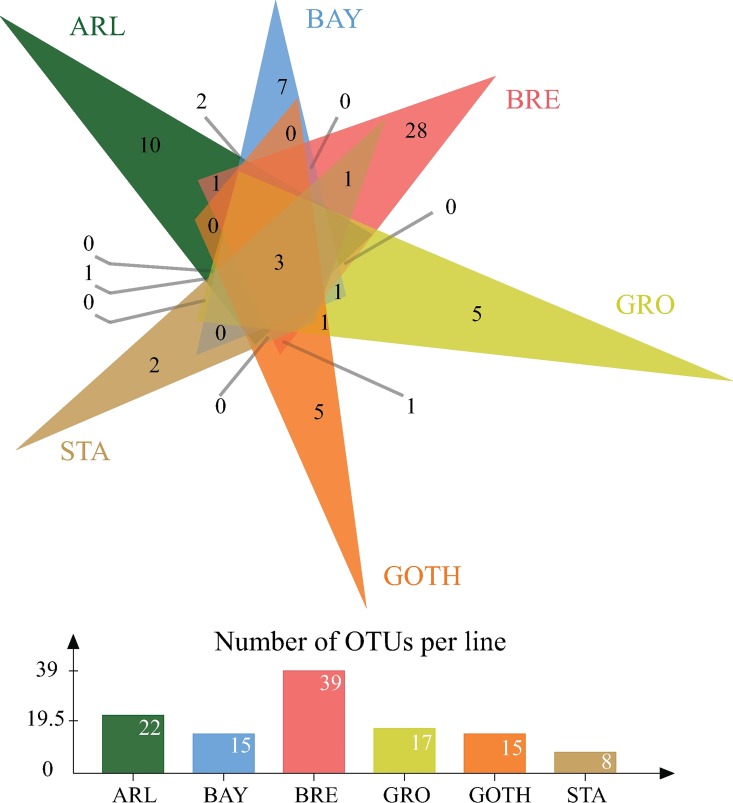
The number of OTUs in the microbiome of six *D*. *melanogaster* lines. The Venn diagram represents OTUs that are shared among the different subsets of lines, or unique to a single line (Venn diagram constructed with jvenn [46]). The barplot represents the total number of OTUs per line. The following line name abbreviations were used: ARL, Arles; BAY, Bayreuth; BRE, Bremen; GOTH, Gotheron; GRO, Groningen; STA, St. Andrews.

The estimated sample coverages (ESC) varied from 0.76 to 0.98 across the libraries (for a detailed description see Table 1). In addition, rarefaction curves are provided (S2 Fig). The richness estimator and the diversity indices (i.e. Chao1 and Shannon, respectively) indicated that the lines differed in their bacterial community richness: BRE had the most diverse and STA the least diverse microbial communities (Table 1). Statistical comparison among lines of the Shannon Index (*F*_*5*,*9*_ = 5.916, *p* = 0.011) and Faith's Phylogenetic Diversity (PD, *F*_*5*,*9*_ = 10.554, *p* = 0.001) indeed revealed significant differences among the lines in alpha diversity (S3 Table). The data on the numbers of OTUs per line were visualized in detail (Fig 2): BRE had 39 OTUs, including 28 that were uniquely found in this line, while STA had only eight OTUs, of which two were unique.

The family Acetobacteriaceae was the most common taxon, present in each replicate of the lines and accounting for 25–95% of the sampled OTUs (Fig 1A). The genus *Wolbachia* was also abundantly found in the samples, except for the line STA (Fig 1B). The BRE line was characterized by a higher abundance of families Planococcaceae, Enterobacteriacea, Staphylococcaceae and Moraxellaceae, which were absent or poorly represented in other lines (Fig 1A). The STA line was associated with low diversity, absence of *Wolbachia* and presence of the family Leuconostocaceae (STA_1) and Micrococcaceae (STA_2). We found only three OTUs to be present across all lines (Fig 2), and these were taxonomically affiliated to the families Acetobacteraceae (two OTUs; *Acetobacter* and unknown genera) and Lactobacillaceae (one OTU; unknown genus).

**Fig 3:**
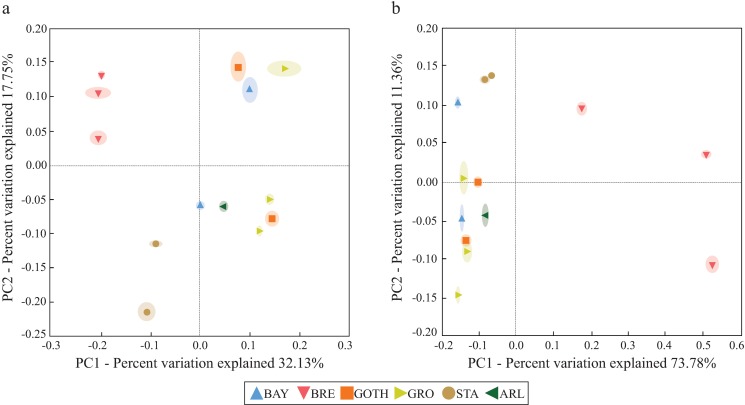
Beta-diversity of bacterial communities associated with *D*. *melanogaster* lines. Bacterial communities are clustered using PCoA of (a) unweighted and (b) weighted UniFrac. The ellipsoid shapes indicate interquartile range (IQR). The percentage of the variation explained by the plotted principal components is indicated on the x- and y-axes. The following line name abbreviations were used: ARL, Arles; BAY, Bayreuth; BRE, Bremen; GOTH, Gotheron; GRO, Groningen; STA, St. Andrews.

Beta-diversity analysis of the *D*. *melanogaster* bacterial communities was performed based on UniFrac distances (Fig 3). The unweighted UniFrac PCoA (Fig 3A) segregated BRE and STA from the other lines in the first axis (Principal component 1, explaining 32.12% of the variation). The second axis (Principal component 2, explained 17.75% of the variation) separated BRE and STA and, in addition, provided evidences for differences across replicates within some *D*. *melanogaster* lines (that is, BAY, GRO and GOTH) (Fig 3A). In the weighted UniFrac plot (Fig 3B), the three BRE replicates segregated apart from the remaining lines (Principal component 1, explaining 73.78% of the variation). The second axis (principal component 2, explaining 11.36% of the variation) showed some small differences across replicates for the lines (see Fig 3B for details). The UPGMA clustering analysis with Jackknife support (S3 Fig) showed a similar pattern and segregated the BRE_3 replicate from other samples. The fact that BRE_3 replicate clustered apart from the remaining two is likely due to differences in taxonomic resolution of the sequences: BRE_3 had a high abundance (i.e. 45%) of sequences taxonomically affiliated to the bacterial genus *Kurthia*, from the Planococcaceae family. While the remaining two BRE replicates also had a high number of sequences affiliated to the family Planococcaceae (Fig 1B), in the case of BRE_1 and BRE_2 the Planococcaceae sequences could not be classified up to the genus level.

### Resistance of *D*. *melanogaster* lines to *A*. *tabida*

**Fig 4:**
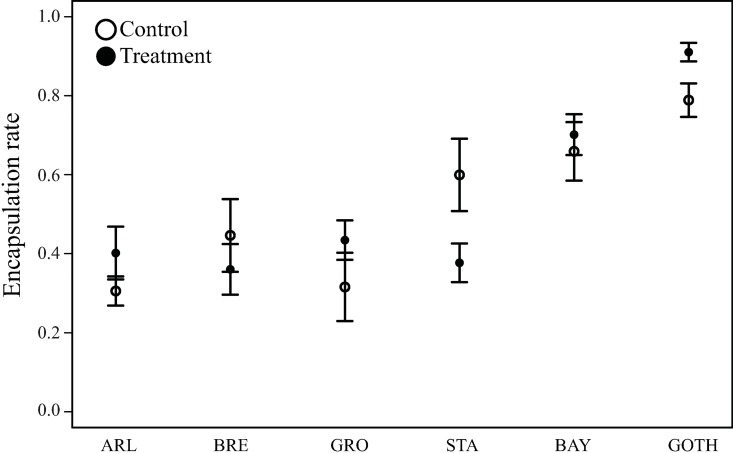
Encapsulation rate (ER) in six *D*. *melanogaster* lines. The circles represent the mean levels of encapsulation ability in antibiotic-treated (black) and control (white) *Drosophila* larvae (with standard errors). The encapsulation rate was measured as the proportion of larvae that successfully encapsulated a parasitoid egg. The following line name abbreviations were used: ARL, Arles; BAY, Bayreuth; BRE, Bremen; GOTH, Gotheron; GRO, Groningen; STA, St. Andrews.

To evaluate whether the microbiome had phenotypic effects on parasitoid resistance, we manipulated the microbiome by treating all lines for three generations with a mixture of antibiotics, followed by rearing the lines for two generations off antibiotics. The phenotypic response to antibiotic treatment differed among the lines (Fig 4). This is also reflected in the statistical analysis: a glm model comparing the effects of antibiotic treatment on resistance among all lines indicated a significant interaction between Line × Treatment (dispersion parameter for quasibinomial family = 3.44, F_5,157_ = 3.563, *p* = 0.0045). A multiple comparison on how the encapsulation rate (ER) was affected differently among lines by antibiotic treatment showed that treated larvae from St. Andrews encapsulated eggs less efficiently (adjusted *p* = 0.0184), while treated larvae of the other lines did not show significant effects of antibiotic treatment on encapsulation. GOTH showed a trend towards increased encapsulation rate (ER) of the parasitoid egg compared to the control group, but this effect was no longer significant after correcting for multiple testing. The antibiotic treatment did not significantly affect pupa-to-adult mortality (dispersion parameter for quasibinomial family = 7.83, glm, F_1,154_ = 1.8748541, not significant). All data and R-scripts of the parasitization experiment are added in the Supplementary Material (S4 Table, S1 File).

### Connecting *Drosophila* resistance to bacterial diversity and abundance

**Fig 5:**
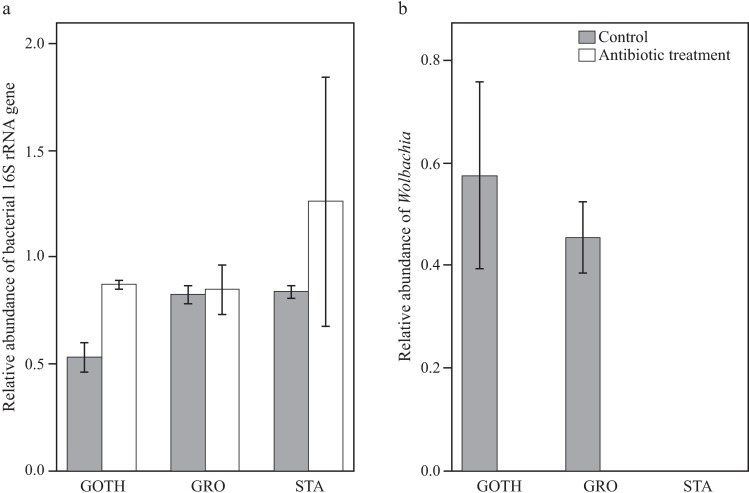
*Wolbachia* and total bacterial load in three *D*. *melanogaster* lines. Relative abundance of bacterial 16S rRNA gene (a) and *gatB* (*Wolbachia*) (b) genes of control (grey) and antibiotic treatment (white) groups in GOTH (Gotheron), GRO (Groningen) and STA (St. Andrews) lines. The values are normalized against TATA-binding protein (*Tbp*), and standard deviations are shown.

In order to quantify *Wolbachia* and total bacterial loads in larvae from control and treated lines, we performed qPCR analysis. The relative abundance of 16S rRNA gene was not significantly different in the treated groups, compared to the control group, nor did it differ between the tested lines (Treatment: F_1,10_ = 2.303, *p* = 0.160, line: F_2,10_ = 1.345, *p* = 0.304, Fig 5). The qPCR data confirmed our sequencing results in showing that STA line had no *Wolbachia* infection. GOTH and GRO of the control groups did not differ in their *Wolbachia* load according to our qPCR results (F_1,6_ = 0.459, *p* = 0.523). An additional qPCR analysis on the remaining three lines (BRE, BAY and ARL) did show differences in *Wolbachia* load among these lines (S4 Fig). After treatment with antibiotics, all lines either lacked (GOTH, GRO, STA, BRE) or had a significantly lower (ARL, BAY) *Wolbachia* load (Fig 5B, S2B Fig).

To verify whether the bacterial communities were altered by the antibiotics treatment and to search for the possible bacterial taxa responsible for the change in resistance, we performed additional sequencing. We sequenced bacterial communities of GOTH and STA lines (control and antibiotic treatment groups), based on the effects antibiotic treatment had on their resistance. We obtained four clone libraries for STA and GOTH (one per control and treatment groups), comprising 87 sequences.

**Fig 6:**
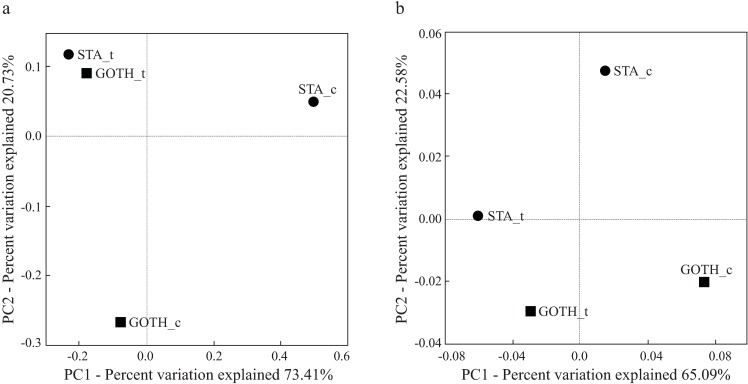
Beta-diversity of bacterial communities associated with two control and two antibiotic-treated lines of *D*. *melanogaster*. Bacterial communities are clustered using PCoA of (a) unweighted and (b) weighted UniFrac. The percentage of the variation explained by the plotted principal components is indicated on the x- and y-axes. Antibiotic treatment and control groups are indicated as letters, ‘t’ and ‘c’ respectively, next to the line abbreviation: GOTH, Gotheron; STA, St. Andrews.

**Fig 7:**
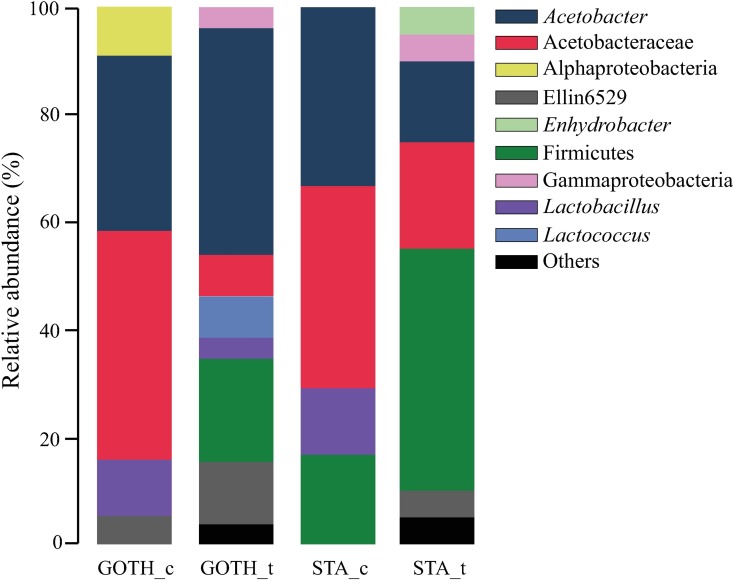
Relative abundance of bacterial taxa in two *D*. *melanogaster* lines, Gotheron (GOTH) and St. Andrews (STA). The relative abundance of taxa was based on taxonomic affiliation of the bacterial 16S rRNA gene fragment. Antibiotic treatment (t) and control (c) treatment are indicated next to the line abbreviation.

Although the total bacterial load of the antibiotic-treated larvae was the same as in the control groups (Fig 5A, S4A Fig), the composition of the microbiome was considerably altered (Fig 6). The sequencing data for GOTH and STA control and antibiotic treatment groups showed that the bacterial communities were dominated by the OTUs belonging to the families Acetobacteraceae and, to a lesser extent, Lactobacillaceae (Fig 7). The UniFrac PCoA grouped the antibiotic-treated samples together (Fig 6), while the control groups of STA and GOTH differed from each other, consistent with the earlier presented microbiome characterization results for all the lines. There was no clear association between any particular OTU and resistance levels against parasitoids.

## Discussion

The first aim of this study was to determine whether host population background affects the microbiome composition. Our results show that six *D*. *melanogaster* lines derived from natural populations differ in their composition and diversity of the bacterial communities, despite being kept on the same standard diet for four consecutive years. This contradicts the earlier findings that *D*. *melanogaster* microbiome is mainly shaped by diet [21,23] and confirms the idea that hosts can either maintain or acquire different bacterial communities [27]. We observed that 2 lines out of 6 tested had distinct and characteristic microbial communities: one relatively species-poor and lacking the common endosymbiont *Wolbachia* (STA), and one relatively species-rich with many unique OTUs (BRE). The other 4 lines (GOTH, BAY, ARL, and GRO) shared intermediate species-richness and more similar microbiomes. The findings in this study show that there is little evidence for a core microbiome in *D*. *melanogaster*, as was also previously suggested elsewhere [21].

What caused the differences among lines in microbiome composition is not solved in our study. Possibly the hosts exercised a certain degree of control over the associated microbiome [23,27,51], and host population genetic differences influenced the microbiome composition [28]. Differences in the host microbial communities could be associated with genetic and physiological differences in the host. The microbiome can be sensitive to slight variations in pH level and availability of oxygen in the gut, or to genetic differences in the host immune system [12,52]. For *Hydra*, a genus of freshwater Cnidarian animals, it was demonstrated that the diverse microbial communities in different species were determined by the differential expression of antimicrobial peptides by the host [51]. Similar to the *Drosophila*-microbiome system, these various *Hydra* species do not have an obligate association with their bacteria, and yet are capable of maintaining a consistent host-specific microbiome.

Alternatively, the observed specificity of the *Drosophila* microbiome among the various lines may arise from the original collections from natural populations. Possibly, the microbiome differences originate from these natural populations, and then persisted over the many generations that the flies were cultured in the lab. Diet is unlikely to have caused the differences, as all lines were reared on identical medium and under identical conditions for over four years. Environmental microbes that grew on the medium are also unlikely to have caused the observed differences among lines, because the medium was heat-sterilized, prepared in batches and distributed among all samples simultaneously. When the founders of the lines had different microbiomes and transmitted these to successive generations, this could provide support for stable and heritable associations of microbiomes, which is one of the requirements for the hologenome theory of evolution. This theory postulates that when the host can maintain a prolonged association with its microbiome, together they form one unit—a holobiont——upon which selection can act [2].

In the ongoing debate about what determines the composition of the *Drosophila* microbiome (diet *versus* taxonomy *versus* random events), it is now clear that various factors play a role and that their relative influences vary across sample types (e.g. instar stages, population background, etc). To fully elucidate the contributing factors would require extensive and carefully designed follow-up experiments, including the characterization of the microbiome of the founders of each line, cross-exposure to the microbiome of different lines, extensive replication within lines (to determine the degree of stochasticity), and laboratory experiments to determine the degree of selection from diet and external conditions. The relative importance of each of the factors probably depends on hosts’ biology, and the role that the microbiome plays in its evolution. Maintaining bacteria that enhance host survival and/or reproduction can give a population a fitness advantage and promote its success. Therefore, the genetic (e.g. immunity, physiology) or ethological (e.g. food preference) mechanisms that promote the growth of the beneficial bacteria can evolve. We already know some of these mutualistic bacteria: for instance, *Lactobacillus plantarum* and acetic acid bacteria enhance growth and starvation resistance [53], exogenous bacteria colonizing the fly’s body surface can contribute to longevity and pheromonal communication among flies [17,19] and *Wolbachia* can enhance stem cell proliferation [54].

The second aim of this study was to investigate the effect of the established microbiome of *D*. *melanogaster* on parasitoid resistance. The natural variation among *D*. *melanogaster* field lines in their resistance to parasites [30,31] and pathogens [26] has been a hallmark for substantial genetic variation in immunity genes. Importantly, our data suggests that an alternative hypothesis also needs to be considered for the natural variation in parasite resistance: perhaps is not only (directly) related to genetic variation among the lines, but it may also be (indirectly) related to the variation in host-microbiome interactions [55].

The ability of the lines to resist the parasitoid *A*. *tabida* was affected in at least one of the lines when treated with antibiotics. After the antibiotic treatment, we quantified and characterized both *Wolbachia* and the total bacterial community abundance and composition. From that, we showed that the manipulation of the microbiome was successful, as demonstrated by substantial shifts in the microbiome of treated flies. Treated flies had not gone through a bottleneck, nor experienced any strong mortality during the antibiotics treatment, which implies that alternations in the host population genetic composition are not to be expected. The observed effects of antibiotic-treatment on parasitoid resistance, therefore, were most likely due to the shifts in the microbiome. While this suggests the influence of the microbiome on parasitoid resistance, only the reintroduction of different OTUs or complete microbiomes could provide a valuable and definitive proof.

We showed that different *Drosophila* lines responded differently to the antibiotic treatment.—STA decreased in their resistance while the other lines did not respond to treatment, with the exception of GOTH, which showed a trend towards increased resistance in response to antibiotic treatment. This finding supports the hypothesis that various bacterial OTUs can have a different function depending on the host genotypic background and/or the total microbiome composition. Therefore removal or acquisition of a certain bacterial taxon could lead to the opposite phenotypic effect (or no effect) in different *Drosophila* populations. For instance, one microbial genus found in *Drosophila*, i.e. *Clostridium*, can modulate host immune response in vertebrates by promoting T_reg_ cell accumulation [56]. Moreover, the genus *Wolbachia* had been reported to enhance *Drosophila* resistance to viruses [13], and both *Wolbachia* and *Spiroplasma* can affect resistance to parasitoids [10,15]. Most studies used an antibiotic treatment to remove endosymbionts followed by a resistance assay. However, we re-emphasize that total bacterial community changes after antibiotic treatment, so it may not be justified to assume that *Wolbachia* or any OTU alone is the cause of the change in the host phenotype [57]. Importantly, in our study, we observed significant changes in the resistance after the antibiotic treatment in the STA line that naturally lacks *Wolbachia* infection. Therefore, we conclude that the intracellular endosymbiont was not the cause of the observed phenotype, i.e. the changed ability to resist the parasitoid.

Although antibiotic treatment itself can also have a negative effect on the host (e.g. [58]), we argue that this was not the case in our study. By keeping the fruit fly cultures off the antibiotics for two generations, we eliminated or reduced the possible negative effect of the treatment on host physiology, and allowed the reestablishment of an altered microbiome, as demonstrated by the recovery in bacterial abundance. This step is often neglected in studies (e.g. [7]), but could jeopardizes the findings when it becomes impossible to make a distinction between the effects of the two factors—stress caused by the antibiotic treatment and the removal or alteration of bacterial types.

We determined resistance to parasitoids based on the flies carrying a melanotic encapsulated parasitoid egg. This measure of resistance ensures that larvae had been parasitized and had successfully defended themselves against the parasitoid. Adult flies that did not carry a capsule were considered to be un-parasitized. In other species of *Drosophila*, larvae can resist parasitoids without forming melanotic capsules (e.g. [14, 15, 59]), which could imply that basing our resistance measure on the presence of visible capsules could underestimate the true level of resistance. In these other *Drosophila* species, however, melanotic capsules have never been found, which reflects the independent evolution of different defense strategies in the *Drosophila* phylogeny [14, 15, 59]. In experiments in which we confirmed parasitizations by behavioural observations, we unambiguously find that larvae that survive parasitization carry visible capsules as adults. We are therefore confident that in *D*. *melanogaster*, the only defense mechanism to survive parasitoid attack relies on melanotic encapsulation.

Finally, in our resistance assays, we observed a relatively high mortality rate among tested *Drosophila* larvae—something that has to be addressed in the future experimental setups. Possibly, the high mortality was caused by super-parasitism, when larvae were parasitized more than once during the assay. We did observe multiple melanotic capsules in several of the adult flies, indicating that superparasitism had indeed occurred. The high mortality may also have led to some slight shifts in ranking of these six lines in terms of resistance, compared to our earlier measurements for these same lines [31]. The high mortality was similar, however, between the antibiotic and control groups, suggesting that it is unlikely that it has caused the observed patterns of altered resistance after antibiotic treatment.

## Conclusions

Our results revealed pronounced differences in the microbiome of genetically differentiated *D*. *melanogaster* lines. Since the tested lines have been maintained on an identical diet for four years, our finding provides an argument against the widely accepted view that diet is the key determinant in *Drosophila*-microbiome system. Our data clearly shows that host population background is an important factor determining bacterial community composition. The question remains, however, what it is in the hosts’ background that caused this strong effect. Part of the variation among lines could be caused by genetic variation (e.g. in immunity and/or gut physiology), and/or part of it may reflect the composition of the microbiome that the founders of these lines acquired in their native environments. Irrespective of which factors caused the microbiome differences in our study, an important implication from our research is that studies that are performed on multiple strains or lines may not only reflect their differences in genotype, but also in the composition of their microbiome, even when these lines were reared in the same laboratory under standardized conditions for many years. Moreover, our data highlights the importance of the *Drosophila* microbiome in shaping host resistance to parasitism, pointing out that *Wolbachia* is not the only determinant of this host phenotype.

## Supporting Information

S1 Fig DGGE analysis of the microbial communities of 4 *D*. *melanogaster* host populations. Lanes 1–2, 15 and 34 (marker), lanes 3–5 (antibiotic treated replicates from Bremen), lanes 6–8 (Bayreuth control), lanes 9–14 (Bayreuth antibiotic treated), lanes 16–18 (Arles control), lanes 19–24 (Arles antibiotic treated), lanes 25–27 (St. Andrews control) and lanes 28–33 (St. Andrews antibiotic treated).
(PDF)

S2 Fig Rarefaction curves of the clone libraries. The original OTU table was rarefied to a depth of 50 sequences per sample (i.e. the lowest in a single sample). One replicate (ARL_2) was excluded due to the low number of sequences. Biological replicates are indicated as numbers next to the line abbreviation: ARL, Arles; BAY, Bayreuth; BRE, Bremen; GOTH, Gotheron; GRO, Groningen; STA, St. Andrews.
(PDF)

S3 Fig Analysis of the β-diversity of the microbiomes of *D*. *melanogaster* lines, represented as UPGMA trees. The jackknife supported (a) unweighted and (b) weighted UniFrac trees. Scale bars indicate distance between line samples in UniFrac units. The abbreviations: ARL, Arles; BAY, Bayreuth; BRE, Bremen; GOTH, Gotheron; GRO, Groningen; STA, St. Andrews.
(PDF)

S4 Fig *Wolbachia* and total bacterial loads. Relative abundance of the 16S rRNA (a) and gat_b (*Wolbachia*) (b) genes of control and antibiotic treatment groups in Arles (ARL), Bayreuth (BAY) and Bremen (BRE) lines based on qPCR analyses. Values are normalized against TATA-binding protein (*Tbp*), and standard errors (SE) are shown. Statistical analyses using a linear mixed effects model for (a) 16S rRNA load: lines: F_2,10_ = 0.030, *p* = 0.970, treatment: F_1,10_ = 0.890, *p* = 0.368 and (b) *Wolbachia* load: lines: F_2,10_ = 6.643, *p* = 0.015, treatment: F_1,10_ = 8.623, *p* = 0.015 and interaction term F_2,10_ = 0.728, *p* = 0.506.
(TIFF)

S1 Table Primers used in the study. (XLSX)

S2 Table OTU table representing *Drosophila* lines microbial composition. Following line name abbreviations were used: ARL, Arles; BAY, Bayreuth; BRE, Bremen; GOTH, Gotheron; GRO, Groningen; STA, St. Andrews.
(XLSX)

S3 Table Alpha diversity comparisons between *D*. *melanogaster* lines. (XLSX)

S4 Table Raw data of the parasitization experiment. (XLSX)

S1 File R-scripts for the statistical analyses of the parasitization experiment. (TXT)
