# Supplementary figures and images for: Bacterial Communities Differ among Drosophila melanogaster Populations and Affect Host Resistance against Parasitoids
Source: PLoS One. 2016 Dec 14;11(12):e0167726. doi: 10.1371/journal.pone.0167726 (PMC5156416; doi:10.1371/journal.pone.0167726)

M BRE\_tr BAY\_c BAY\_tr M ARL\_c ARL\_tr STA\_c STA\_tr M

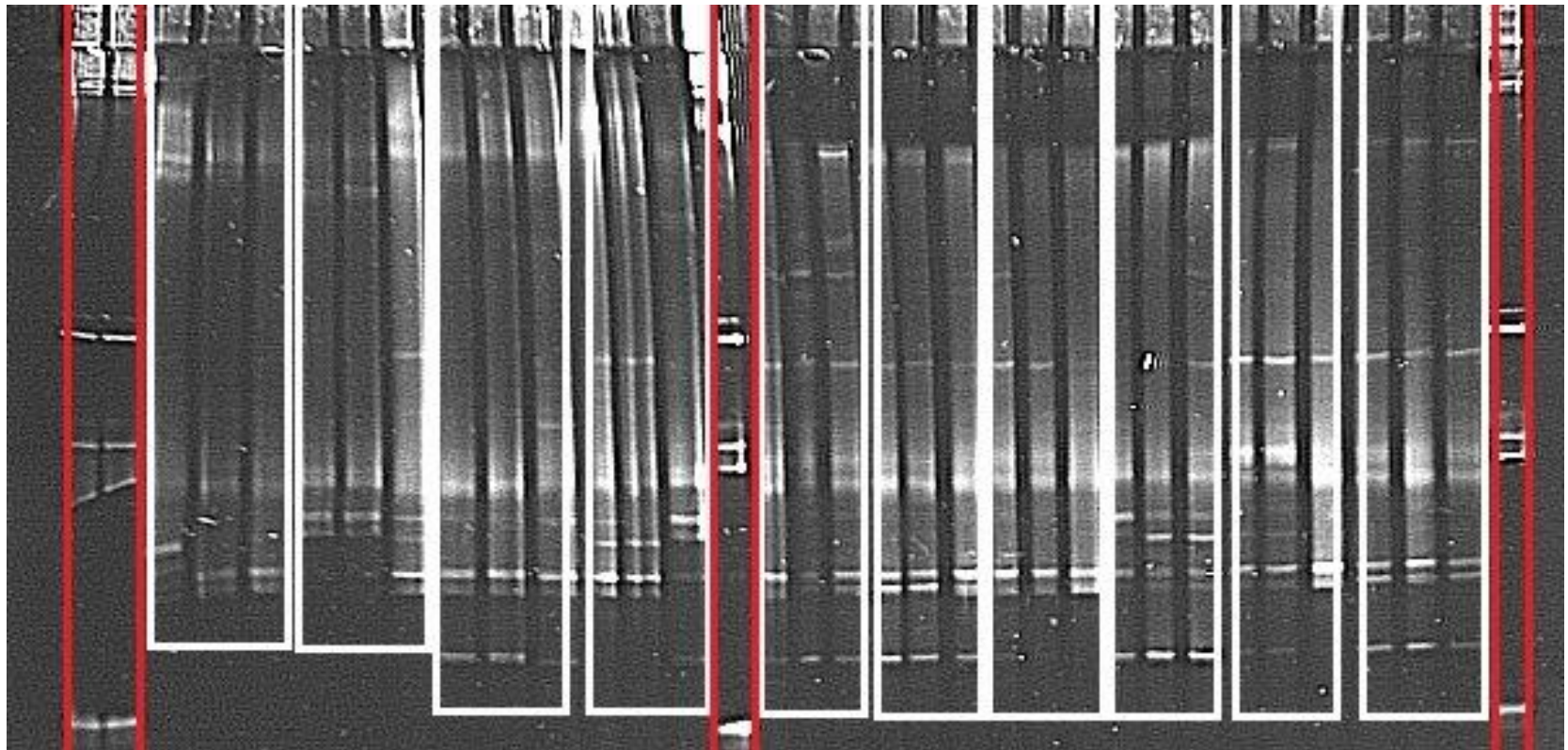

Supplement: S1 Fig — Lanes 1–2, 15 and 34 (marker), lanes 3–5 (antibiotic treated replicates from Bremen), lanes 6–8 (Bayreuth control), lanes 9–14 (Bayreuth antibiotic treated), lanes 16–18 (Arles control), lanes 19–24 (Arles antibiotic treated), lanes 25–27 (St. Andrews control) and lanes 28–33 (St. Andrews antibiotic treated). (PDF) [file pone.0167726.s001.pdf]

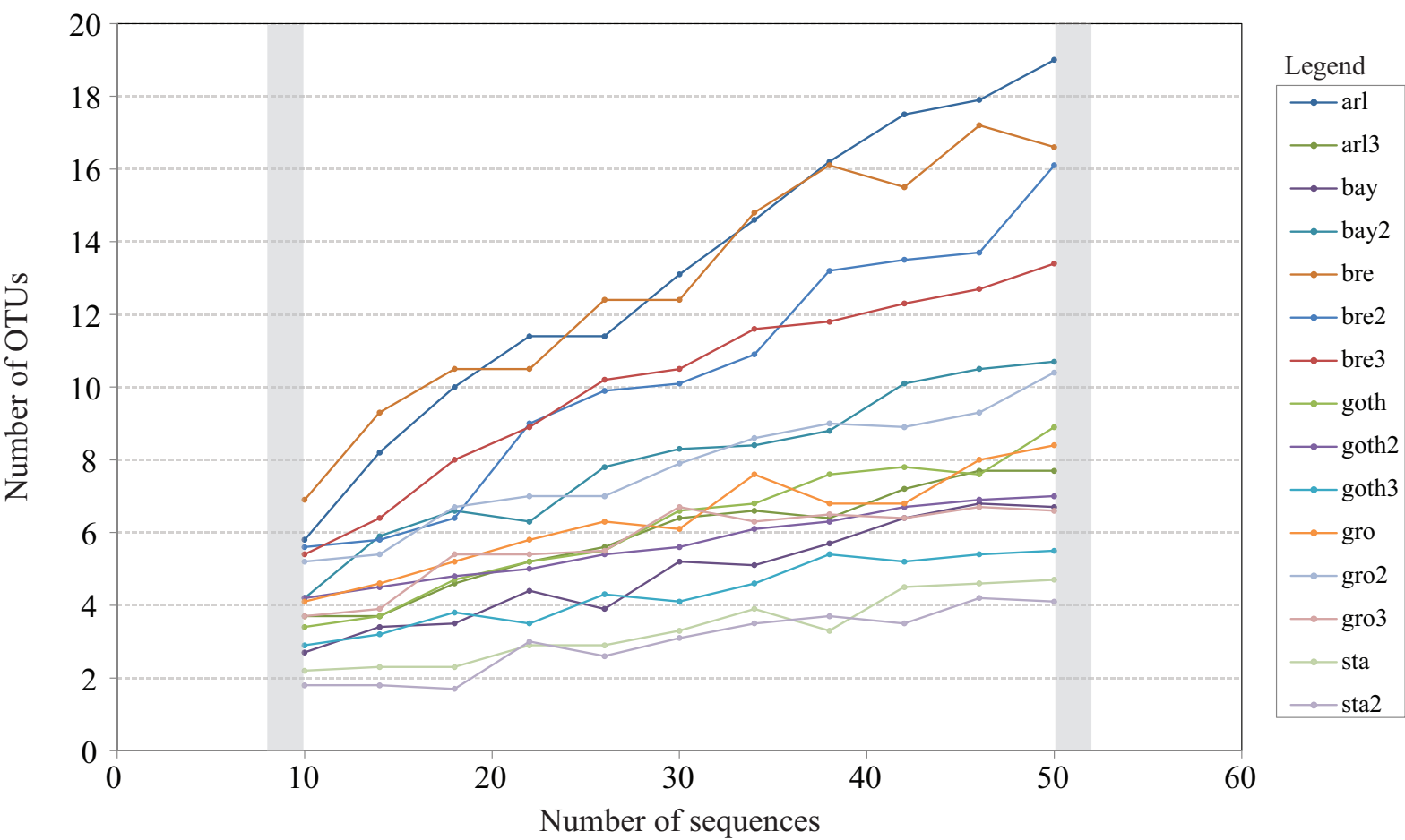

Supplement: S2 Fig — The original OTU table was rarefied to a depth of 50 sequences per sample (i.e. the lowest in a single sample). One replicate (ARL_2) was excluded due to the low number of sequences. Biological replicates are indicated as numbers next to the line abbreviation: ARL, Arles; BAY, Bayreuth; BRE, Bremen; GOTH, Gotheron; GRO, Groningen; STA, St. Andrews. (PDF) [file pone.0167726.s002.pdf]

**a**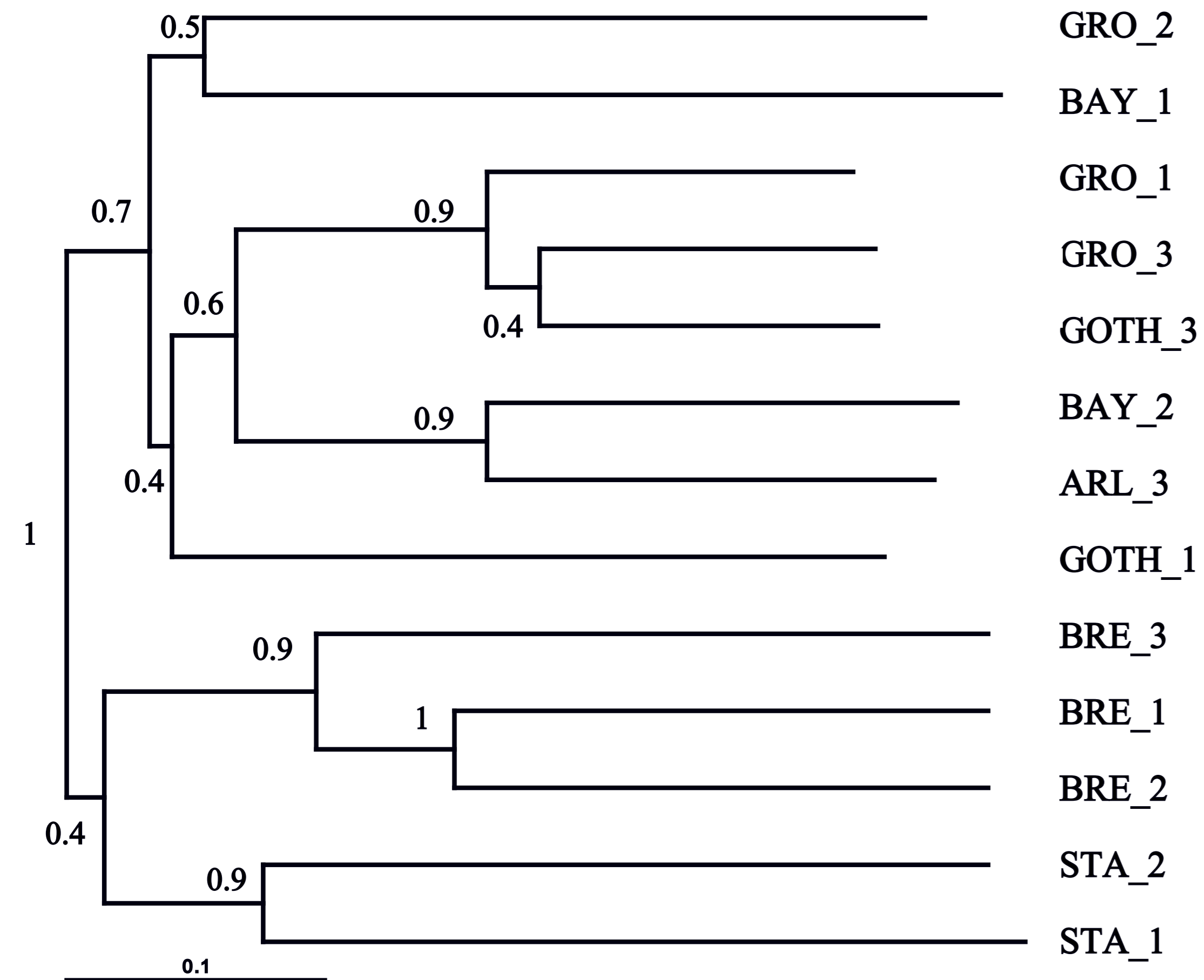**b**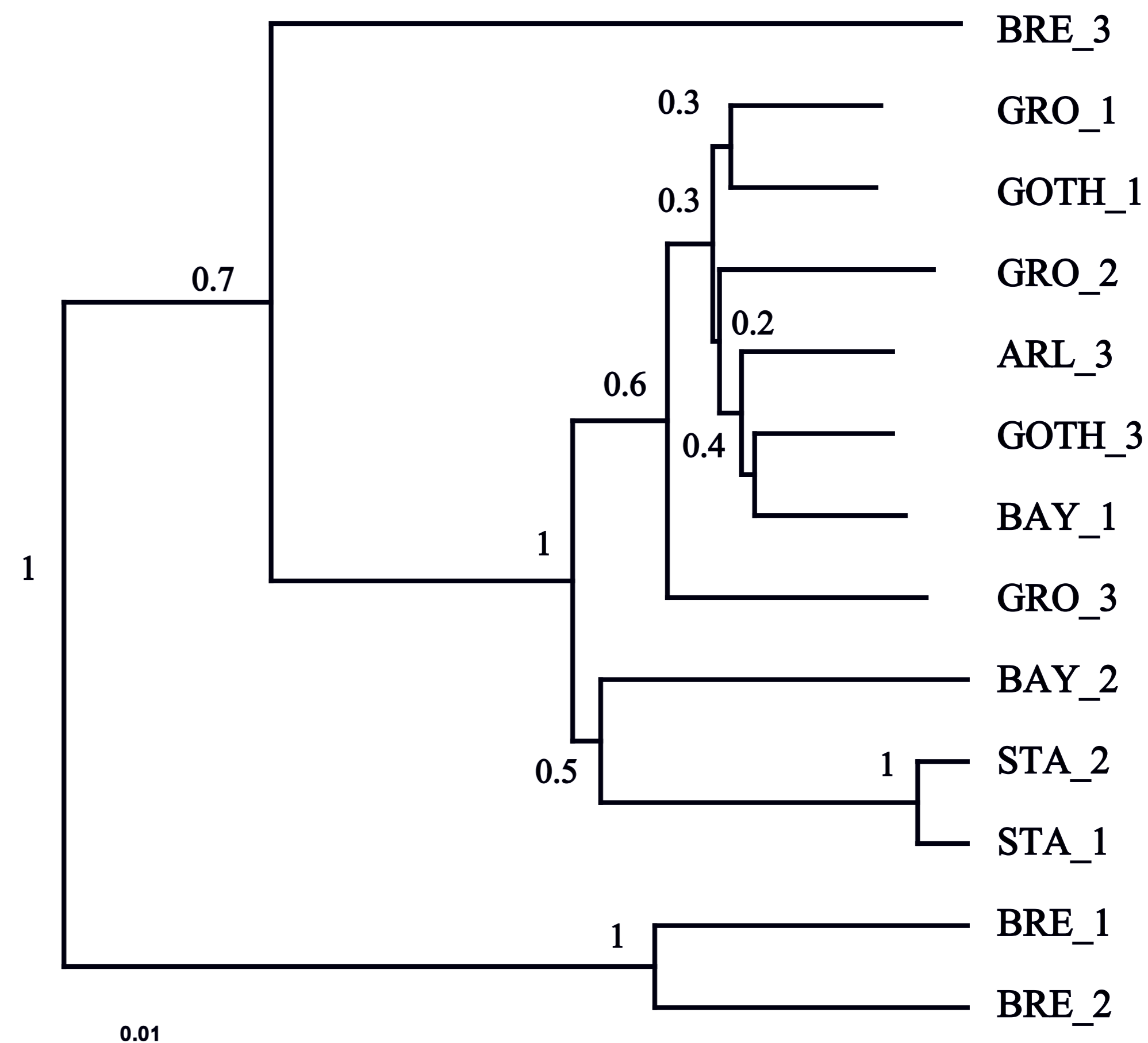

Supplement: S3 Fig — The jackknife supported (a) unweighted and (b) weighted UniFrac trees. Scale bars indicate distance between line samples in UniFrac units. The abbreviations: ARL, Arles; BAY, Bayreuth; BRE, Bremen; GOTH, Gotheron; GRO, Groningen; STA, St. Andrews. (PDF) [file pone.0167726.s003.pdf]

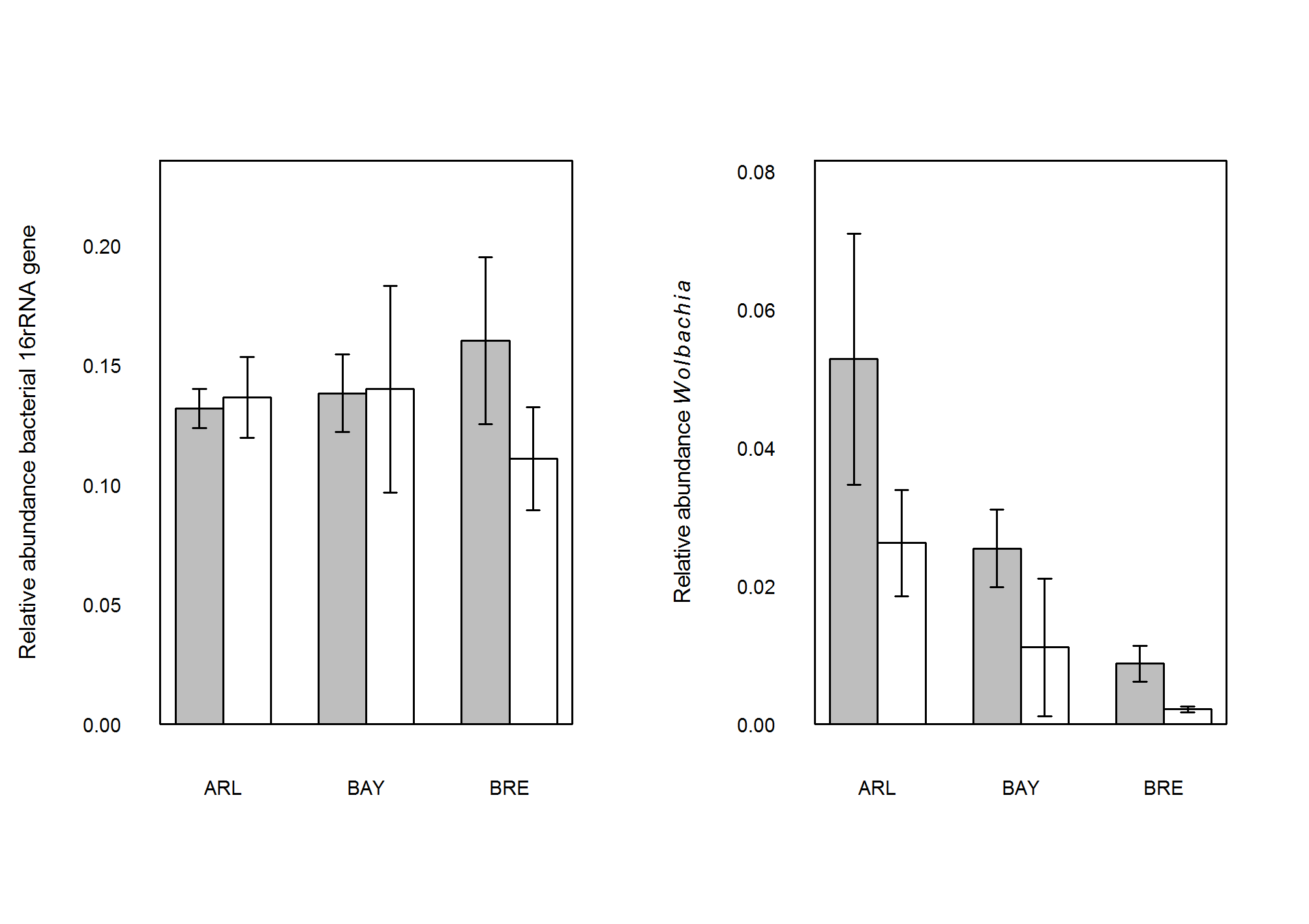

Supplement: S4 Fig — Relative abundance of the 16S rRNA (a) and gat_b (Wolbachia) (b) genes of control and antibiotic treatment groups in Arles (ARL), Bayreuth (BAY) and Bremen (BRE) lines based on qPCR analyses. Values are normalized against TATA-binding protein (Tbp), and standard errors (SE) are shown. Statistical analyses using a linear mixed effects model for (a) 16S rRNA load: lines: F2,10 = 0.030, p = 0.970, treatment: F1,10 = 0.890, p = 0.368 and (b) Wolbachia load: lines: F2,10 = 6.643, p = 0.015, treatment: F1,10 = 8.623, p = 0.015 and interaction term F2,10 = 0.728, p = 0.506. (TIFF) [file pone.0167726.s004.tiff]
